# Supplementary material for: Draft genome of the Northern snakehead, Channa argus
Source: Gigascience. 2017 Mar 2;6(4):1–5. doi: 10.1093/gigascience/gix011 (PMC5530311; doi:10.1093/gigascience/gix011)

Manuscript

Click here to download Manuscript Revised manuscript NEW.docx

Draft genome of the Northern snakehead, *Channa argus*

Jian Xu[1,2]†, Chao Bian[2,3,4]†, Kunci Chen[5]†, Guiming Liu[6], Yanliang Jiang[1], Qing Luo[5], Xinxin You[2,3], Wenzhu Peng[1,7], Jia Li[3], Yu Huang[3], Yunhai Yi[3], Chuanju Dong[1,8], Hua Deng[9], Songhao Zhang[1], Hanyuan Zhang[1], Qiong Shi[2,3,10]*, Peng Xu[1,2,7]*

[1] Key Laboratory of Aquatic Genomics, Ministry of Agriculture, CAFS Key Laboratory of Aquatic Genomics and Beijing Key Laboratory of Fishery Biotechnology, Chinese Academy of Fishery Sciences, Beijing, 100141, China

[2] BGI Research Center for Aquatic Genomics, Chinese Academy of Fishery Sciences, Shenzhen, 518083, China.

[3] Shenzhen Key Lab of Marine Genomics, Guangdong Provincial Key Lab of Molecular Breeding in Marine Economic Animals, BGI, Shenzhen, 518083, China.

[4] Centre of Reproduction, Development and Aging, Faculty of Health Sciences, University of Macau, Taipa, Macau, China;

[5] Pearl River Fisheries Research Institute, Chinese Academy of Fishery Sciences, Guangzhou, 510380, China.

[6] CAS Key Laboratory of Genome Sciences and Information, Beijing Institute of Genomics, Chinese Academy of Sciences, Beijing, 100029, China.

[7] Fujian Collaborative Innovation Center for Exploitation and Utilization of Marine Biological Resources, Xiamen University, Xiamen, 361102, China.

[8] College of Fishery, Henan Normal University, Xinxiang, 453007, China.

[9]Research Institute of Forestry Policy and Information,Chinese Academy of Forestry, Wanshoushan, Haidian District, Beijing 100091, China.

[10] Laboratory of Aquatic Genomics, College of Ecology and Evolution, School of Life Sciences, Sun Yat-Sen University, Guangzhou, 510275, China.

†Contributed equally to this work.

*Correspondence: xupeng77@xmu.edu.cn, shiqiong@genomics.cn

Email addresses: xuj@cafs.ac.cn (JX), bianchao@genomics.cn (CB), chenkunci@aliyun.com (KC), liugm@big.ac.cn (GL), jiangyl@cafs.ac.cn (YJ), luoqing@prfri.ac.cn (QL), youxinxin@genomics.cn (XY), 695705687@qq.com (WP), lijia1@genomics.cn (JL), huangyu@genomics.cn (HY), cjd1989@126.com (CD), denghua@caf.ac.cn (HD), 378479568@qq.com (SZ), zhanghanyuan@cafs.ac.cn (HZ), shiqiong@genomics.cn (QS), xupeng77@xmu.edu.cn (PX)

Abstract

Background: The Northern snakehead (*Channa argus*), a member of the Channidae family of the Perciformes, and is an economically important freshwater fish native to East Asia. In North America, it has become notorious as an intentionally released invasive species. Its ability to breathe air with gills and migrate short distances over land makes it a good model for bimodal breath research. Therefore, recent research has focused on the identification of relevant candidate genes. Here, we performed whole genome sequencing of *C. argus* to construct its draft genome, aiming to offer useful information for further functional studies and identification of target genes related to its unusual facultative air breathing.

Findings: We assembled the *C. argus* genome with a total of 140.3 Gigabases (Gb) of raw reads, which were sequenced using the Illumina HiSeq2000 platform. The final draft genome assembly was approximately 615.3 million bases (Mb), with a contig N50 of 81.4 kb and scaffold N50 of 4.5 Mb. The identified repeat sequences account for 18.9% of the whole genome. 19,877 protein-coding genes was predicted from the genome assembly, with an average of 10.5 exons per gene.

Conclusion: We generated a high-quality draft genome of *C. argus*, which will provide a valuable genetic resource for further biomedical investigations of this economically important teleost fish.

Keywords: *Channa argus*, Genome assembly, Annotation, Gene prediction

Data description

Introduction of *C. argus*

The Northern snakehead (*Channa argus*) is a special snakehead fish mainly cultivated in Asia and Africa for food, especially in China with an annual production of about 510,000 tons (worth ~1.6 billion US dollars) (Figure 1). Genetic degradation caused by inbreeding of *C. argus* cultivation has led to higher susceptibility to diseases. Furthermore, *C. argus* is considered as a serious invasive species in North America, due to its wide-range diet, parental care, rapid colonization and expansion [1]. *C. argus* has a specialized aerial breathing organ, the suprabranchial chamber, which facilitates its aquatic–aerial bimodal breathing. Because of its aggressive status in ecosystem of rivers, lakes and ponds, and little consumption of the *C. argus* in America for food, this leads to threats of to the balance ecosystems. For both economic and ecological consideration, it is vital to develop genomic resources for further genetic breeding studies or ecological research. So far, the genome sequence of *C. argus* has not been reported, and hence in our current study we performed genome sequencing, assembly, and annotation of this teleost species.

C. argus genome sequencing on the Illumina platform

Genomic DNA was extracted from blood sample of a single female *C. argus* (Fishbase ID: 4799) using Qiagen GenomicTip100 (Qiagen, Hilden, USA). The fish was obtained from the Pearl River Fisheries Research Institute, Chinese Academy of Fishery Sciences, Guangzhou, China. A whole-genome shotgun sequencing strategy was applied, and short-insert libraries (180-bp, 500-bp and 800-bp) and long-insert libraries (3-kb and 5-kb) were constructed using the standard protocol provided by Illumina (San Diego, USA). Paired-end sequencing with a 2 x 100-bp read length was performed on the short-insert and long-insert libraries using the Illumina HiSeq2000 platform. In total, we generated about 140.3 Gb of raw reads, including 33.0, 36.9, 17.4, 26.5 and 26.5 Gb of reads from the 180-bp, 500-bp, 800-bp, 3-kb and 5-kb libraries. After removal of low-quality and redundant reads, we obtained about 138.2

Gb of clean data for further *de novo* assembling of the *C. argus* genome.

Estimation of *C. argus* genome size and sequencing coverage

All the cleaned reads were subjected to 17-mer frequency distribution analysis. As the total number of *k*-mers was about 5.90×10^{10} and the peak of *k*-mers at a depth of 88, the genome size of *C. argus* was calculated to be 670.4 Mb using the following formula: genome size = *k*-mer_number / peak_depth. Therefore, the sequencing coverage was found to be ~ 124.5× based on the estimated genome size.

De novo genome assembly and quality assessment

For whole genome assembly, SOAPdenovo2 [3] was used with optimized parameters (-K 75) to construct contigs and original scaffolds by using the reads from short-insert libraries. All reads were then mapped onto contigs for scaffold construction by utilizing the paired-end information of long-insert libraries. Some intra-scaffold gaps were filled by local software using read-pairs in which one end uniquely mapped to a contig and the other end was located within a gap. Finally, a draft *C. argus* genome of 615.3 Mb was assembled, with a contig N50 size of 81.4 kb and a scaffold N50 size of 4.5 Mb (Table 1).

Subsequently, CEGMA (Core Eukaryotic Genes Mapping Approach) software [4] (version 2.3) with 248 conserved Core Eukaryotic Genes (CEGs) was utilized to evaluate completeness of genes. Our results demonstrated that the generated genome assembly covered 242 of the 248 CEG sequences, suggesting a high level of completeness within the genome assembly. Alongside this, we also used BUSCO (version 1.22) [5] (the representative vertebrate gene set containing 3,023 single-copy genes that are highly conserved in vertebrates) software to assess the quality of the generated genome assembly. The assessment demonstrated that the BUSCO value is 82.9%, containing C: 66% [D: 1.4%], F: 16%, M: 17%, n: 3,023 (C: complete [D: duplicated], F: fragmented, M: missed, n: genes), suggesting a high quality of the generated assembly.

Repeat sequence within the *C. argus* genome assembly

To analyze the *C. argus* genome, we employed Tandem Repeats Finder [6] (version 4.04) with core parameters set as “Match = 2, Mismatch = 7, Delta = 7, PM = 80, PI = 10, Minscore = 50, and MaxPerid = 2000” to identify tandem repeats. Simultaneously, RepeatModeler (version 1.04) and LTR_FINDER [7] were utilized to construct a *de novo* repeat library with default parameters. Subsequently, we used RepeatMasker [8] (version 3.2.9) to map our assembled sequences on the Repbase TE (version 14.04) [9] and the *de novo* repeat libraries to search for known and novel transposable elements (TEs). In addition, the TE-related proteins were annotated by using RepeatProteinMask software [8] (version 3.2.2). In summary, the total identified repeat sequences accounted for 18.94% of the *C. argus* genome (Table 2). Among them, long interspersed nuclear elements (LINE) were the most abundant type of repeat sequences, and occupy 8.92% of the whole genome.

Gene annotation

Gene annotation of the *C. argus* genome was conducted using several approaches, including transcriptome-based prediction, *de novo* prediction and homology-based prediction. RNA-seq datasets of pooled 13 tissues were obtained from our previous work [10]. We mapped these RNA reads onto our genome assembly using TopHat1.2 software [11], and then we employed Cufflinks (version 2.2.1) [12] to predict the gene structures. Furthermore, we performed Augustus (version 2.5.5) [13], GlimmerHMM (version 3.0.1) [14] and GenScan (version 1.0) [15] softwares for *de novo* prediction on the repeat-masked *C. argus* genome assembly. The protein sequences of zebrafish (*Danio rerio*) [16], Japanese puffer (*Fugu rubripes*) [17], medaka (*Oryzias latipes*) [18], spotted green pufferfish (*Tetraodon nigroviridis*) [19] (above 5 species were downloaded from Ensembl release 75), blue spotted mudskipper (*Boleophthalmus boddarti*) [20] and golden arowana (*Scleropages formosus*) [21] were mapped on the *C. argus* genome using TblastN with e-value $\leqslant$ 1e-5. Subsequently, Genewise2.2.0 software [22] was employed to predict the potential gene structures on

all alignments. Finally, the above three datasets were integrated to yield a comprehensive and non-redundant gene set using GLEAN (https://sourceforge.net/projects/glean-gene/) [23] with several filter steps (removing partial sequences or genes shorter than 150 bp or prematurely terminated/frame-shifted genes). The final total gene set was composed of 19,877 genes, with an average of 10.5 exons per gene (Table 1).

Construction of gene families and phylogenetic tree

We downloaded the protein sequences of zebrafish [17], Japanese puffer [18], stickleback (*Gasterosteus aculeatus*) [24], spotted green pufferfish [20], medaka [19] from the Ensembl Core database (release 75), and we also obtained the protein sequences of Asian seabass (*Lates calcarifer*) [25], blue spotted mudskipper [21] and golden arowana [22] from their corresponding ftp websites, respectively. The consensus proteome set of the above eight species and snakehead fish were filtered to remove those protein sequences less than 50 amino acids, and resulted in a dataset of 190,566 protein sequences, which was used as the input file for OrthoMCL [26] to construct gene families. A total of 17,954 OrthoMCL families were built utilizing an effective database size of 190,566 sequences for all-to-all BLASTP strategy with an E-value of 1e-5 and a Markov Chain Clustering (MCL) default inflation parameter. We further identified 24 gene families that were specific in the snakehead fish (Figure 2a).

Subsequently, we selected 1,918 single-copy (only one gene from each species) orthogroups from the above-mentioned 9 teleost species. We used MUSCLE (version 3.8.31) [27] to align the protein sequences from the 1,918 orthogroups, respectively. We also converted protein alignments to their corresponding CDS alignments using an in-house perl script. All the translated CDS sequences were then combined into one “supergene” for each species. Non-degenerated sites (4D) extracted from the supergenes were then joined into new sequence of each species to construct a phylogenetic tree (Figure 2b) using MrBayes [28] (Version 3.2, with the GTR+gamma

model).

Conclusion

We report the first whole genome sequencing, assembly, and annotation of the Northern snakehead (*Channa argus*). The final draft genome assembly is approximately 615.3 Mb, accounting for 91.8% of the estimated genome size (670.4 Mb). We also predicted 19,877 protein-coding genes from the generated assembly.

The draft genome assembly will be valuable resource for genetic breeding, environmental DNA detection of invasive species, and biological studies on this economically important teleost fish. Based on these genomic data, researchers will be able to develop genetic markers for further quantitative trait locus (QTL) and genome-wide association studies (GWAS) on growth traits. These markers will also be very useful for DNA barcoding in screening invasive *C. argus* for ecological protection.

Table 1. Summary of the *Channa argus* genome assembly and annotation.

Genome assembly	
Contig N50 size (kb)	8.1
Contig number (>100bp)	29,146
Scaffold N50 size (Mb)	4.5
Scaffold number (>100bp)	5,297
Total length (Mb)	615.3
Genome coverage (X)	224.6
The longest scaffold (bp)	18,736,006
**Genome annotation**	
Protein-coding gene number	19,877

Mean transcript length (kb)	16.5
Mean exons per gene	10.5
Mean exon length (bp)	175.0
Mean intron length (bp)	1,537.3

Table 2. The detailed classification of repeat sequences of *Channa argus*

Type	Repbase TEs Length (bp)	Repbase TEs % in genome	TE Protiens Length (bp)	TE Protiens % in genome	*De novo* Length (bp)	*De novo* % in genome	Combined TEs Length (bp)	Combined TEs % in genome
DNA	17984515	2.92	6784728	1.10	25663752	4.17	35435946	5.76
LINE	16799343	2.73	17563763	2.85	54890557	8.92	60651866	9.86
SINE	4512385	0.73	0	0	6672552	1.08	9026285	1.47
LTR	4421728	0.72	3031607	0.49	24144657	3.92	26983318	4.39
Other	8125	0.001	0	0	0	0	8125	0.001
Unknown	0	0	0	0	9413375	1.53	9413375	1.53
Total	41585442	6.76	27363267	4.45	103162115	16.77	116545270	18.94

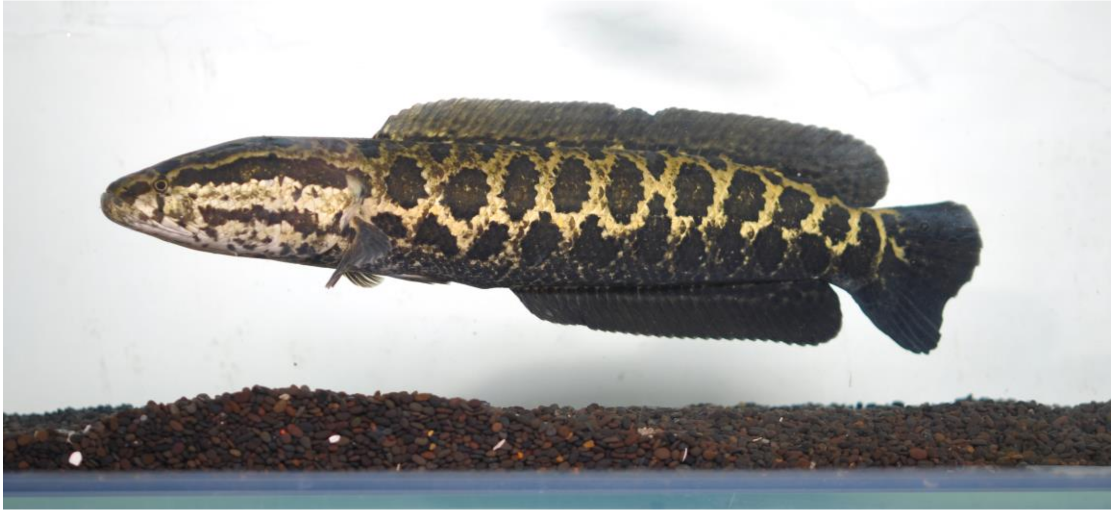

Figure 1. The Northern snakehead fish, *Channa argus*

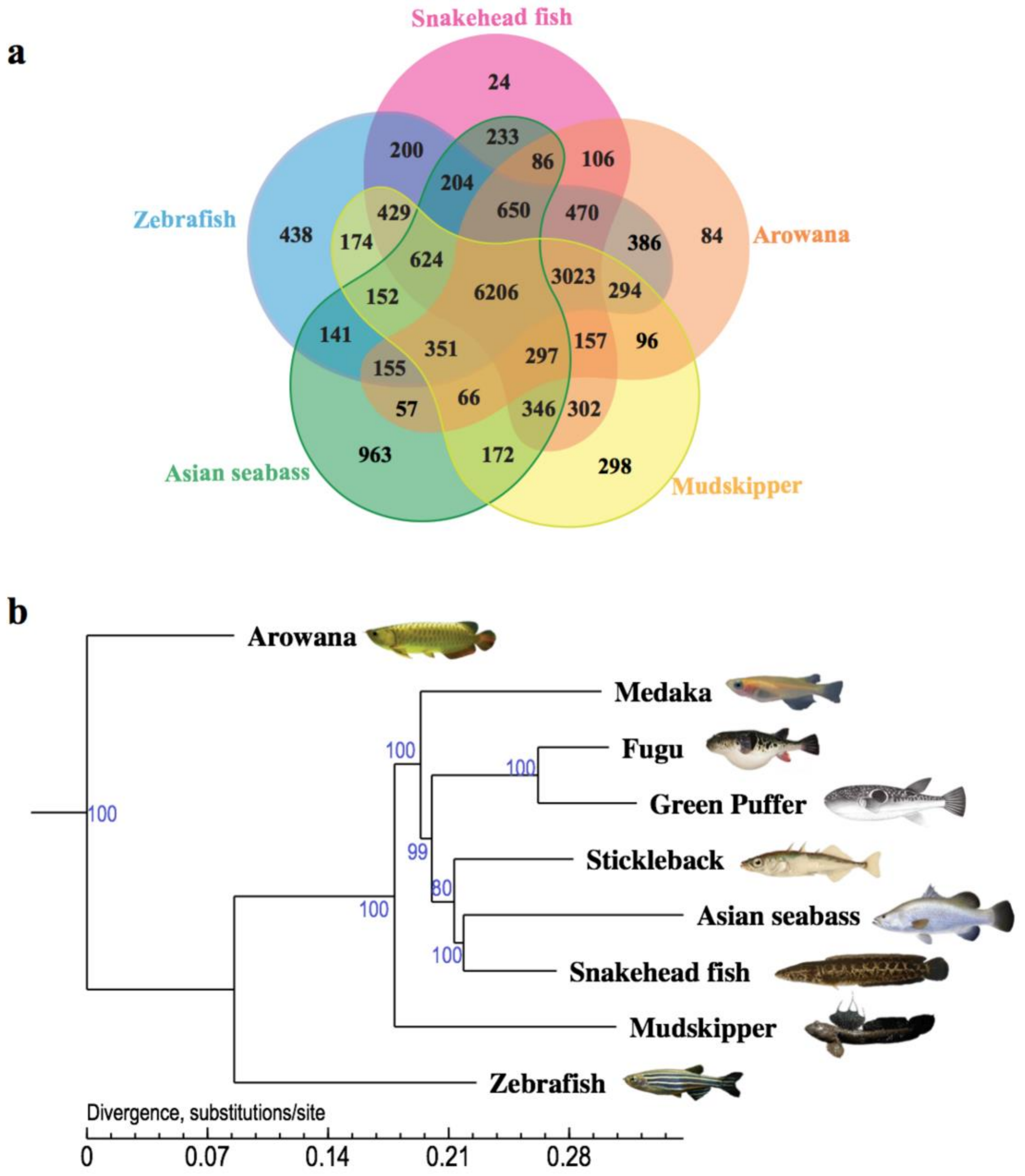

Figure 2. Genome evolution. (a) Orthologous gene families across five fish genomes (Snakehead fish, Zebrafish, Asian seabass, Mudskipper and Arowana). (b) Phylogeny of ray-finned fishes (the arowana as the outgroup species).

Availability of supporting data

The raw sequencing reads of all libraries have been deposited at NCBI (SRP078899). Further supporting data are available in the *GigaScience* database, GigaDB [29].

Abbreviations

CDS: Coding DNA sequence; CEG: Core Eukaryotic Gene; Gb: Gigabase; LINE: long interspersed nuclear element; TE: transposable element.

Authors' contributions

PX designed the study. JX, CB, GL, JL, HD, YH, YX and QS assembled and annotated the genome. CB and YY performed the evolution analysis. JX, YJ, XY, QL and HZ analyzed the data. WP, CD, SZ and KC collected the sample and prepared the quality control. JX, CB, QS and PX wrote the manuscript. QS and PX participated in discussions and provided advice. All authors read and approved the final manuscript.

Acknowledgements

This work was supported by Central Public-interest Scientific Institution Basal Research Fund, CAFS (No. 2015C005, No. 2016HY-JC0301), the National Natural Science Foundation of China (No. 31422057, No.31402291), the National Infrastructure of Fishery Germplasm Resources of China (No. 2017DKA30470), Special Project on the Integration of Industry, Education and Research of Guangdong Province (No. 2013B090800017), Quality Inspection Programs of Scientific Research Project (No. 2015IK246), and Shenzhen Special Program for Future Industrial Development (No. JSGG20141020113728803).

Competing interests

The authors declare that they have no competing interests.

References

1. Jiang Y, Feng S, Xu J, Zhang S, Li S, Sun X, Xu P: **Comparative transcriptome analysis between aquatic and aerial breathing organs of Channa argus to reveal the genetic basis underlying bimodal respiration**. *Mar Genomics*.
2. Marcais G, Kingsford C: **A fast, lock-free approach for efficient parallel counting of occurrences of k-mers**. *Bioinformatics* 2011, **27**(6):764-770.
3. Luo R, Liu B, Xie Y, Li Z, Huang W, Yuan J, He G, Chen Y, Pan Q, Liu Y *et al*: **SOAPdenovo2: an empirically improved memory-efficient short-read de**

novo assembler. *GigaScience* 2012, **1**(1):18.

4. Parra G, Bradnam K, Korf I: **CEGMA: a pipeline to accurately annotate core genes in eukaryotic genomes**. *Bioinformatics* 2007, **23**(9):1061-1067.
5. Simao FA, Waterhouse RM, Ioannidis P, Kriventseva EV, Zdobnov EM: **BUSCO: assessing genome assembly and annotation completeness with single-copy orthologs**. *Bioinformatics* 2015, **31**(19):3210-3212.
6. Benson G: **Tandem repeats finder: a program to analyze DNA sequences**. *Nucleic acids research* 1999, **27**(2):573-580.
7. Xu Z, Wang H: **LTR_FINDER: an efficient tool for the prediction of full-length LTR retrotransposons**. *Nucleic acids research* 2007, **35**(Web Server issue):W265-268.
8. Tarailo-Graovac M, Chen N: **Using RepeatMasker to identify repetitive elements in genomic sequences**. *Current protocols in bioinformatics / editoral board, Andreas D Baxevanis [et al]* 2009, **Chapter 4**:Unit 4 10.
9. Jurka J, Kapitonov VV, Pavlicek A, Klonowski P, Kohany O, Walichiewicz J: **Repbase Update, a database of eukaryotic repetitive elements**. *Cytogenetic and genome research* 2005, **110**(1-4):462-467.
10. Jiang Y, Feng S, Xu J, Zhang S, Li S, Sun X, Xu P: **Comparative transcriptome analysis between aquatic and aerial breathing organs of Channa argus to reveal the genetic basis underlying bimodal respiration**. *Mar Genomics* 2016:DOI: 10.1016/j.margen.2016.1006.1002.
11. Trapnell C, Pachter L, Salzberg SL: **TopHat: discovering splice junctions with RNA-Seq**. *Bioinformatics* 2009, **25**(9):1105-1111.
12. Trapnell C, Williams BA, Pertea G, Mortazavi A, Kwan G, van Baren MJ, Salzberg SL, Wold BJ, Pachter L: **Transcript assembly and quantification by RNA-Seq reveals unannotated transcripts and isoform switching during cell differentiation**. *Nature biotechnology* 2010, **28**(5):511-515.
13. Stanke M, Steinkamp R, Waack S, Morgenstern B: **AUGUSTUS: a web server for gene finding in eukaryotes**. *Nucleic acids research* 2004, **32**(Web Server issue):W309-312.
14. Majoros WH, Pertea M, Salzberg SL: **TigrScan and GlimmerHMM: two open source ab initio eukaryotic gene-finders**. *Bioinformatics* 2004, **20**(16):2878-2879.
15. Cai Y, Gonzalez JV, Liu Z, Huang T: **Computational systems biology methods in molecular biology, chemistry biology, molecular biomedicine, and biopharmacy**. *BioMed research international* 2014, **2014**:746814.
16. Howe K, Clark MD, Torroja CF, Torrance J, Berthelot C, Muffato M, Collins JE, Humphray S, McLaren K, Matthews L *et al*: **The zebrafish reference genome sequence and its relationship to the human genome**. *Nature* 2013, **496**(7446):498-503.
17. Aparicio S, Chapman J, Stupka E, Putnam N, Chia JM, Dehal P, Christoffels A, Rash S, Hoon S, Smit A *et al*: **Whole-genome shotgun assembly and analysis of the genome of Fugu rubripes**. *Science* 2002, **297**(5585):1301-1310.

18. Kasahara M, Naruse K, Sasaki S, Nakatani Y, Qu W, Ahsan B, Yamada T, Nagayasu Y, Doi K, Kasai Y *et al*: **The medaka draft genome and insights into vertebrate genome evolution**. *Nature* 2007, **447**(7145):714-719.
19. Jaillon O, Aury JM, Brunet F, Petit JL, Stange-Thomann N, Mauceli E, Bouneau L, Fischer C, Ozouf-Costaz C, Bernot A *et al*: **Genome duplication in the teleost fish Tetraodon nigroviridis reveals the early vertebrate proto-karyotype**. *Nature* 2004, **431**(7011):946-957.
20. You X, Bian C, Zan Q, Xu X, Liu X, Chen J, Wang J, Qiu Y, Li W, Zhang X *et al*: **Mudskipper genomes provide insights into the terrestrial adaptation of amphibious fishes**. *Nature communications* 2014, **5**:5594.
21. Bian C, Hu Y, Ravi V, Kuznetsova IS, Shen X, Mu X, Sun Y, You X, Li J, Li X *et al*: **The Asian arowana (Scleropages formosus) genome provides new insights into the evolution of an early lineage of teleosts**. *Scientific reports* 2016, **6**:24501.
22. Birney E, Clamp M, Durbin R: **GeneWise and Genomewise**. *Genome research* 2004, **14**(5):988-995.
23. Elsik CG, Mackey AJ, Reese JT, Milshina NV, Roos DS, Weinstock GM: **Creating a honey bee consensus gene set**. *Genome biology* 2007, **8**(1):R13.
24. Jones FC, Grabherr MG, Chan YF, Russell P, Mauceli E, Johnson J, Swofford R, Pirun M, Zody MC, White S *et al*: **The genomic basis of adaptive evolution in threespine sticklebacks**. *Nature* 2012, **484**(7392):55-61.
25. Vij S, Kuhl H, Kuznetsova IS, Komissarov A, Yurchenko AA, Van Heusden P, Singh S, Thevasagayam NM, Prakki SR, Purushothaman K *et al*: **Chromosomal-Level Assembly of the Asian Seabass Genome Using Long Sequence Reads and Multi-layered Scaffolding**. *PLoS genetics* 2016, **12**(4):e1005954.
26. Li L, Stoeckert CJ, Jr., Roos DS: **OrthoMCL: identification of ortholog groups for eukaryotic genomes**. *Genome research* 2003, **13**(9):2178-2189.
27. Edgar RC: **MUSCLE: multiple sequence alignment with high accuracy and high throughput**. *Nucleic acids research* 2004, **32**(5):1792-1797.
28. Ronquist F, Teslenko M, van der Mark P, Ayres DL, Darling A, Hohna S, Larget B, Liu L, Suchard MA, Huelsenbeck JP: **MrBayes 3.2: efficient Bayesian phylogenetic inference and model choice across a large model space**. *Systematic biology* 2012, **61**(3):539-542.
29. Xu, J; Bian, C; Chen, K; Liu, G; Jiang, Y; Luo, Q; You, X; Peng, W; Li, J; Huang, Y; Yi, Y; Dong, C; Deng, H; Zhang, S; Zhang, H; Shi, Q; Xu, P (2017): **Supporting data for the draft genome of the Northern snakehead, Channa argus.** GigaScience Database. http://dx.doi.org/10.5524/100279

 Click here to download Figure Northern snakehead.jpg

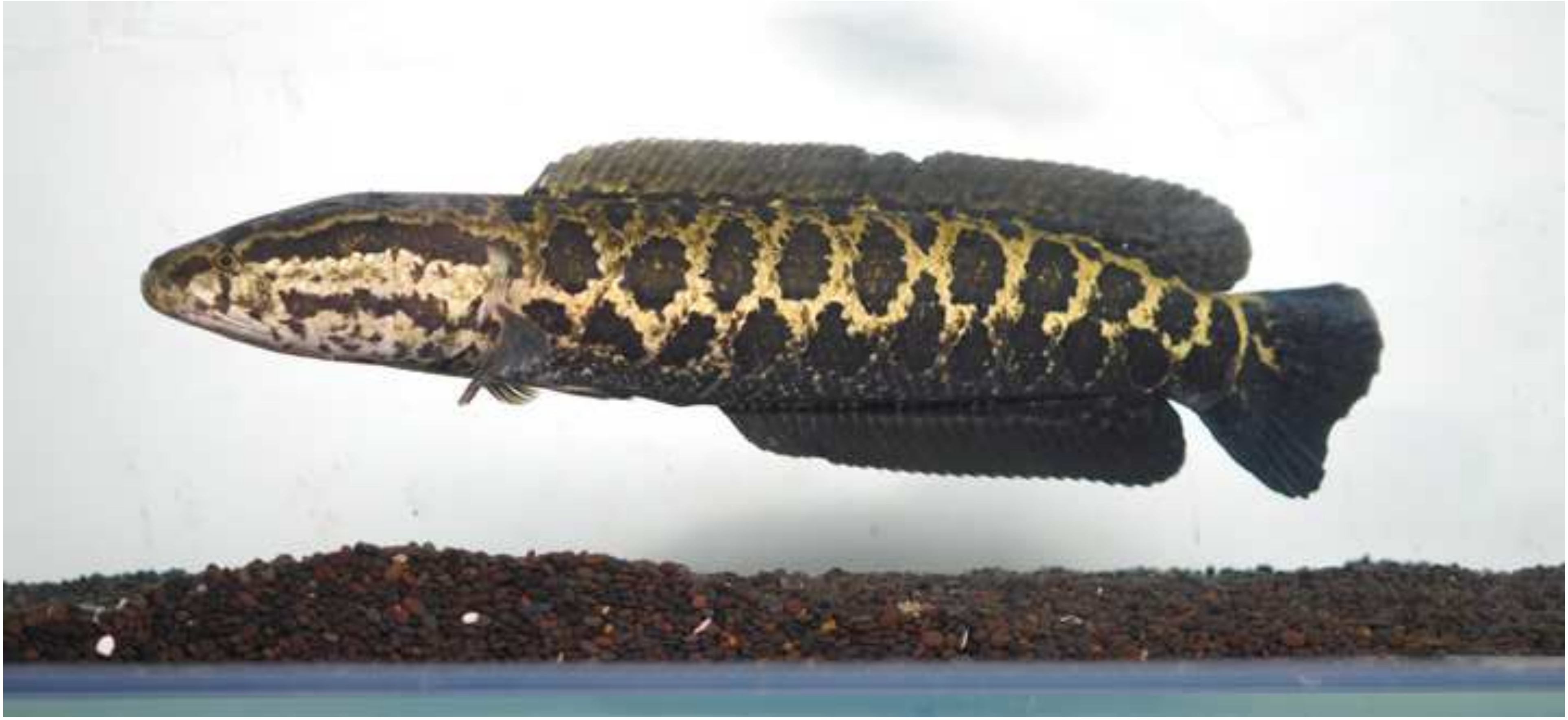

Supplement: GIGA-D-16-00078_Revision_2.pdf [file gix011_GIGA-D-16-00078_Revision_2.pdf]
