# Supplementary material for: Draft genome of the Northern snakehead, Channa argus
Source: Gigascience. 2017 Mar 2;6(4):1–5. doi: 10.1093/gigascience/gix011 (PMC5530311; doi:10.1093/gigascience/gix011)
Supplement: Response_to_reviewer_comments_Original_Submission.pdf [file gix011_Response_to_reviewer_comments_Original_Submission.pdf]

Dear editor,

Thanks for your kind help to provide the comments of reviewers.
We have revised our manuscript according to your suggestions and the comments of reviewers one by one.
Classification of repeat sequences was summarized in the new Table 2.
By the way, it took such a long time for us to submit our point-by-point responses, because we performed more sequencing (500-bp and 800-bp libraries). Therefore, quality of the new genome assembly has been increased remarkably. For example, the scaffold N50 is increased to 4.5 Mb from the previous 1.1 Mb.

Best regards,
Qiong Shi, PhD, Professor
BGI
Shenzhen 518083
China

P.S. Our point-by-point responses are provided as follows for your consideration.

Reviewer reports:
Reviewer #1: The manuscript "Draft genome of the Northern snakehead, Channa argus" is an important contribution to the scientific community working on comparative teleost genomics. It is submitted with the purpose of releasing this valuable dataset to the public, and holds no claim to solve any specific scientific question, but rather put up possibilities for the future use of this dataset.

###General comments
The sequencing and assembly method is clearly explained, with some minor exceptions (see specific comments below).
The authors have also done a good job on identifying repeated structures (LTRs, LINEs, TEs etc), yet this reviewer would like to see the results of these analyses summarized in a table or figure.

Answer: Thanks for your nice suggestion. The detailed information of repeat sequences is summarized in the new Table 2.

The annotation of genes in the C. argus genome also appear thorough, although newer software and pipelines could have been utilized (i.e MAKER (Holt and Yandell 2011 and Campbell et al. 2014). That being said, this reviewer do not see any purpose in redoing these analyses at this point, as it would only serve to delay this dataset from being released to the public.
Finally, the authors also present a phylogenetic tree, placing the C. argus at the expected position within the Percomorphacea. The methodology used for this analysis is, in this reviewers opinion, not "state-of-the-art". However, as this phylogenetic inference makes no claim to be anything than a confirmation of the quality of the data, this is acceptable in this regard.

Answer: Thanks for the assessment of our gene set and phylogenetic data. As the editor mentioned, our current Data Note will focus on the primary genome data with few analyses so as to improve the speed of public availability. Deep analyses will be initiated soon for different purposes.

The authors should, however, address the language in this manuscript, and have it proof-read by a native English speaker. Especially this reviewer finds the use of was and were to be incorrect, and the use of "subsequently" and (especially) "simultaneously" should be reconsidered.

Answer: Thanks for your kind help. We have corrected language errors as more as possible with help from an American scientist.

###Specific comments
Lines 40-43. The argumentation for why this species genome will be invaluable in researching "post-

operative pain and discomfort" is lost on this reviewer. If this is the case, please make this clearer to the reader. These two lines also appear repetitive, and should be rephrased.

Answer: Thanks for your good comments. The phrase “post-operative pain and discomfort” was deleted in the revised abstract to make the sentence clearer. Meanwhile, a short description of the wound healing function was added to the Introduction section (lines 74-75).

Lines 64-66. Some change of wording and some better flow should be considered. This reviewer cannot see the contradictions depicted here with "However" and "Contrary to this". Please rephrase to make this clear.

Answer: Thanks for your instructive comments. This sentence has been rephrased and the conjunction was replaced with “Meanwhile”(lines 66-69).

Line 72. The optioning of "media and movies" is hardly relevant in this context. Please rephrase / remove.

Answer: The irrelevant words were removed in the current revision.

Lines 75 -76. Final statement should include the result, not a statement of what the authors do for work. Please rephrase to correct english.

Answer: Thanks for the comment. This is in the Introduction section, so we just described the content briefly without detailed results. More details are provided in the followed sections respectively.

Line 100. Please explain the parameters and how they were optimized.

Answer: Due to the parameters of Soapdenovo2 (especially the –K parameter) are empirical, we performed massive Soapdenovo analyses with a series of K parameters (25, 35, 45, 55, 65 and 75). After a careful evaluation, we selected the most optimized parameter -K 75 for further assembly.

Lines 101-103. These statements need rephrasing and are also quire repetitive.

Answer: Thanks for your instructions. These repetitive sentences were removed in the revised manuscript.

Line 104. What is this "local software"? If this was used in the analyses please make it public by depositing this in a GitHub repository and refer to the URL in the end of the ms.

Answer: This local software, named as “Gapcloser”, was widely used for many genome works, such as channel catfish [1], mudskippers [2], and P. xylostella [3]. We uploaded this software to the FTP of Gigascience.

1. Chen X et al: High-quality genome assembly of channel catfish, Ictalurus punctatus. GigaScience 2016, 5(1):39.
2. You X et al: Mudskipper genomes provide insights into the terrestrial adaptation of amphibious fishes. Nature communications 2014, 5:5594.
3. You M et al: A heterozygous moth genome provides insights into herbivory and detoxification. Nature genetics 2013, 45(2):220-225.

Line 113. Please also state the version of BUSCO used.

Answer: The BUSCO version (1.22) was added according to your instruction (line 117 in the revised manuscript).

Line 114. There is also a "actinopterygii" gene set available. For future purposes please contact the authors of BUSCO to get a copy of this set (currently not publicly available yet).

Answer: Thanks for this creative suggestion, which will be applied for our further investigations.

Line 115. Consider replacing "widely present" with "highly conserved"

Answer: Thanks for the comment. Related words were changed (line 119 in the revised manuscript).

Lines 130-132. As stated above, please make a table (or figure) for these results including the percentages of all types of repeats (i.e output from RepeatModeler). Also please make sure RepeatModeler is spelled correctly.

Answer: Thanks for your suggestions. The TE results were summarized in the new Table 2. Meanwhile, the spelling of RepeatModeler was corrected (line 128) in the revised manuscript.

Lines 139-140, 147 and 159. Please specify version used for the listed software.

Answer: Related version of the listed software was provided according to your instruction (lines 129-133 &142-146 in the revised manuscript).

Lines 141-144 and 153-156. Please add scientific names to the species used (first time)

Answer: Thanks for your nice comments. These scientific names of fish species were added in the revised manuscript (lines 146-150 & 161-165).

Line 143. What species does "Ensemble release 75" refer to? What about the rest?

Answer: The protein sequences of Zebrafish, Japanese puffer, Medaka and spotted green pufferfish were downloaded from the Ensembl release 75, while the protein sequences of blue spotted mudskipper and golden arowana were downloaded from NCBI. Please see more details on lines 146-151 of the revised manuscript.

Line 145 and 162. Please make sure the e-value is reported in the correct way.

Answer: It was corrected.

Line 157. Please restrain from claiming ownership of the fish. Rephrase.

Answer: Thanks for the comments. This sentence was rephrased (lines 83-85).

Line 163. Gene families or Orthogroups?

Answer: They are orthogroups. Please see the revision on line 176.

Line 167-168. Need to rephrase so that it is clear that a "multiple-alignment" was performed for each, and not just multiple (as in "an unspecified number of") alignments.

Answer: Thanks for your correction. We modified this sentence based on your instruction (lines 176-179 in the revised manuscript).

Line 170. This "in house perl script" should also be made available on GitHub or similar depositories.

Answer: Yes, we uploaded this perl script to the FTP of Gigascience for public availability.

Lines 179-181. The argumentation here needs some rephrasing. Be more explicit in how this will be valuable and why this arguments are valid.

Answer: Thanks for your suggestion. These sentences were revised (lines 191-200).

Line 184. "will be also" needs to be corrected.

Answer: Thanks for the comment. The sentence was corrected.

Line 186-187. This reviewer find it hard to believe that there is a direct effect of C.argus on wound healing. Please rephrase / make more clear, especially as reference [29] is not readily available, nor understandable for many scientists.

Answer: Thanks for your suggestions. Actually, only few papers reported the potential effect of C. argus on wound healing. The following paper was also cited for easy availability.
[2] Mustafa A et al. Albumin and Zinc content of snakehead fish (Channa striata) extract and its role in health. International Journal of Science and Technology, 2012, 1(2):1-8.

Reviewer #2: Review for the manuscript number GIGA-D-16-00078:

In this manuscript Xu and colleagues reports the draft genome of the Northern snakehead (Channa argus), an economically important freshwater fish that is mainly distributed in Asia and Africa. However, this fish was recently released in North America where, because of its aggressive behavior, represents a dangerous invasive species. Notably, this fish has a specialized breathing organ that allows aerial breathing and thus survival for days on land. Considering its economic relevance, the particular additional breathing mode and the role in affecting ecosystem balances, the draft genome of the snakehead will be a valuable resource to facilitate future biomedical, ecological and in general research targeting phenotypic traits.
However, even though I believe that the amount of sequence data allowed to generate a robust draft genome, the authors did not analyze the genome deeply, and they did not address any specific scientific question. In the light of this main concern and of other minor points, I think that this manuscript needs a substantial revision before to be considered for publication in Gigascience, a journal that publishes robust studies relying on 'big data'.

Major comments:

Nowadays the production of million/billion sequences in not a limiting factor anymore, therefore the publication of a genome is not interesting enough without a deep data mining to provide a detailed characterization of the genome and, more importantly, to address relevant biological questions. From a physiological point of view, for example, with its bimodal breathing mode, the Northern snakehead represents a great system to investigate the gene/gens pathways related to respiratory metabolisms. Using other published genomes, with a comparative genomics approach, it would be interesting to analyze what is peculiar of this species. I believe that this type of analysis, and this is only an example, will make this study more interesting for a broader audience.
The manuscript suffers from lack of clarity. Many sentences would benefit from being re-written and in general all the manuscript should be proofread before to be resubmitted to this or to any other journal.

Answer: Thanks you very much for the suggestions. As the editor mentioned, our current Data Note will focus on the primary genome data with few analyses so as to improve the speed of public availability. Deep analyses will be initiated soon for different purposes.

Minor comments:
Line 48: 86.0-Gb ◊ 86 Gb
Line 65: "Contrary" to what?
Lines 69-72: This sentence is poorly written, please revise it.

Answer: Thanks for your nice advice. Changes were made according to your suggestions.

Line 78-90: The whole paragraph is weak as several details are missing:
- Which reagents/kit did you use for DNA extraction?

Answer: Qiagen GenomicTip100 (Qiagen, Hilden, DE) were used for extraction of genomic DNA. Please see the revision on line 83.

- Which Illumina kit was used to construct the genomic libraries? Did you use PCR free kit for the paired end libraries? And for the Mate pair? Did you use the Nextera kit? This information is important and the way in which the Mate pair have been processed/filtered needs to be detailed.

Answer: Sorry, we couldn't provide more details about the methods and kits for library construction, since they are commercial secretes of BGI.

- I guess that the read length is 2x100 bp. Please state it.

Answer: Yes, this information was changed (line 88).

- How many reads for paired read and how many for Mate pair reads? (translate in Gb too).

Answer: Thanks for your instructive comments. In fact, we generated about 86.0 gigabases (Gb) of raw reads containing 33.0 Gb, 26.5 Gb and 26.5 Gb of reads from 180-bp, 3-kb and 5-kb libraries. In our recent work, we added more sequencing data. Please check the detailed information on lines 90-93.

- For the further genome assembly, are short insert-reads and long-insert reads used separately in different steps? Please add more details.

Answer: Yes, short insert-reads were used for constructing the contigs, but the reads from long-insert libraries were utilized for linking the contigs to scaffolds. Please find the clarifications on lines 104-107.

- Which software and what criteria have been used to process low-quality and redundant reads?

Answer: We already uploaded this in-house script to the FTP of Gigascience. The optimized parameters were set as "-g 1 -o clean -M 2 -f 0".

Line 101: What are these "optimized parameters"?

Answer: Due to the parameters of Soapdenovo2 (especially the –K parameter) are empirical, we performed massive Soapdenovo analyses with a series of K parameters (25, 35, 45, 55, 65 and 75). After a careful evaluation, we selected the most optimized parameter -K 75 for further assembly.

Line 120: For the TE analysis, more details about other class of TEs should be provided. How many known and novel TEs were detected in the genome?

Answer: Thanks for your suggestion. The detailed repeat statistics were summarized in the new Table 2.

Line 112: more than 97.6% is vague. Why don't just use the number of covered CEG sequences?

Answer: Yes, the sentence was revised according to your instruction. Please see more details on line 116 of the revised manuscript (covered 242 of 248 CEG).

Line 141-145: Why the protein sequences from Stickleback and Asian seabass, the two closest species, were

not included in this analysis?

Answer: Thanks for your nice suggestion. However, because stickleback and Asian seabass are the closest species for the snakehead, the homolog results from these two species would be covered by the transcriptome and de novo annotations. That is to say, using some more distant species can predict a more completed gene set. That is why we excluded the sequences of these two fishes from this analysis.

Line 163-164: The text is not consistent with the Figure 1a. In the text, the authors state that there are 963 species-specific gene families in the Northern snakehead genome, while the corresponding number, as I can see in the Venn diagram, is 24.

Answer: Sorry for the mistake. We corrected this error in the revised manuscript (line 172).
