# Supplementary material for: Draft genome of the Northern snakehead, Channa argus
Source: Gigascience. 2017 Mar 2;6(4):1–5. doi: 10.1093/gigascience/gix011 (PMC5530311; doi:10.1093/gigascience/gix011)
Supplement: Response_to_reviewer_comments_Revision_1.pdf [file gix011_Response_to_reviewer_comments_Revision_1.pdf]

Dear editor,

Thanks for your kind help to provide the comments of reviewers.
We have revised our manuscript according to your suggestions and the comments of reviewers.

The larger image of the species will be sent to you by e-mail.

Best regards,
Qiong Shi, PhD, Professor
BGI
Shenzhen 518083
China

P.S. Our responses are provided as follows.

The authors have adequately addressed my concerns. Furthermore, adding more sequences, they produced a very high quality draft genome of the Northern snakehead providing a valuable resource for future genomics studies for this and other closely related species. My only concern is about the language used, therefore I would suggest the authors get editing help from someone with full professional proficiency in English to improve the quality of the manuscript.
Answer: Thanks for your suggestion. We obtained help from a Chinese expert who had been in the USA for 9 years. Please see the polished sentences on lines 81, 84-85, 89, 120-121, 134-135, 162 and 195 of the revised manuscript with the yellow color to highlight these changes.
