# Supplementary material for: Draft genome of the Northern snakehead, Channa argus
Source: Gigascience. 2017 Mar 2;6(4):1–5. doi: 10.1093/gigascience/gix011 (PMC5530311; doi:10.1093/gigascience/gix011)
Supplement: Reviewer_1_Report_(Original_Submission).pdf [file gix011_Reviewer_1_Report_(Original_Submission).pdf]

Reviewer Report

Title: "Draft genome of the Northern snakehead, Channa argus"

Version: Original Submission **Date:** 9/16/2016

Reviewer name: Martin Malmstrøm

Reviewer Comments to Author:

The manuscript "Draft genome of the Northern snakehead, Channa argus" is an important contribution to the scientific community working on comparative teleost genomics. It is submitted with the purpose of releasing this valuable dataset to the public, and holds no claim to solve any specific scientific question, but rather put up possibilities for the future use of this dataset.

###General comments

The sequencing and assembly method is clearly explained, with some minor exceptions (see specific comments below).

The authors have also done a good job on identifying repeated structures (LTRs, LINEs, TEs etc), yet this reviewer would like to see the results of these analyses summarized in a table or figure.

The annotation of genes in the C. argus genome also appear thorough, although newer software and pipelines could have been utilized (i.e MAKER (Holt and Yandell 2011 and Campbell et al. 2014). That being said, this reviewer do not see any purpose in redoing these analyses at this point, as it would only serve to delay this dataset from being released to the public.

Finally, the authors also present a phylogenetic tree, placing the C. argus at the expected position within the Percomorphacea. The methodology used for this analysis is, in this reviewers opinion, not "state-of-the-art". However, as this phylogenetic inference makes no claim to be anything than a confirmation of the quality of the data, this is acceptable in this regard.

The authors should, however, address the language in this manuscript, and have it proof-read by a native english speker. Especially this reviewer finds the use of was and were to be incorrect, and the use of "subsequently" and (especially) "simultaneously" should be reconsidered.

###Specific comments

Lines 40-43. The argumentation for why this species genome will be invaluable in researching "post-operative pain and discomfort" is lost on this reviewer. If this is the case, please make this clearer to the reader. These two lines also appear repetitive, and should be rephrased.

Lines 64-66. Some change of wording and some better flow should be considered. This reviewer con not see the contradictions depicted here with "However" and "Contrary to this". Please rephrase to make this clear.

Line 72. The optioning of "media and movies" is hardly relevant in this context. Please rephrase / remove.

Lines 75 -76. Final statement should include the result, not a statement of what the authors do for work. Please rephrase to correct english.

Line 100. Please explain the parameters and how they were optimized.

Lines 101-103. These statements need rephrasing and are also quire repetitive.

Line 104. What is this "local software"? If this was used in the analyses please make it public by depositing this in a GitHub repository and refer to the URL in the end of the ms.

Line 113. Please also state the version of BUSCO used.

Line 114. There is also a "actinopterygii" gene set available. For future purposes please contact the authors of BUSCO to get a copy of this set (currently not publicly available yet).

Line 115. Consider replacing "widely present" with "highly conserved"

Lines 130-132. As stated above, please make a table (or figure) for these results including the percentages of all types of repeats (i.e output from RepeatModeler). Also please make sure RepeatModeler is spelled correctly.

Lines 139-140, 147 and 159. Please specify version used for the listed software.

Lines 141-144 and 153-156. Please add scientific names to the species used (first time)

Line 143. What species does "Ensemble release 75" refer to? What about the rest?

Line 145 and 162. Please make sure the e-value is reported in the correct way.

Line 157. Please restrain from claiming ownership of the fish. Rephrase.

Line 163. Gene families or Orthogroups?

Line 167-168. Need to rephrase so that it is clear that a "multiple-alignment" was performed for each, and not just multiple (as in "an unspecified number of") alignments.

Line 170. This "in house perl script" should also be made available on GitHub or similar depositories.

Lines 179-181. The argumentation here needs some rephrasing. Be more explicit in how this will be valuable and why this arguments are valid.

Line 184. "will be also" needs to be corrected.

Line 186-187. This reviewer find it hard to believe that there is a direct effect of C.argus on wound healing. Please rephrase / make more clear, especially as reference [29] is not readily available, nor understandable for many scientists.

Level of Interest

Please indicate how interesting you found the manuscript: An article of importance in its field

Quality of Written English

Please indicate the quality of language in the manuscript: Needs some language corrections before being published

Declaration of Competing Interests

Please complete a declaration of competing interests, considering the following questions:

- Have you in the past five years received reimbursements, fees, funding, or salary from an organisation that may in any way gain or lose financially from the publication of this manuscript, either now or in the future?
- Do you hold any stocks or shares in an organisation that may in any way gain or lose financially from the publication of this manuscript, either now or in the future?
- Do you hold or are you currently applying for any patents relating to the content of the manuscript?
- Have you received reimbursements, fees, funding, or salary from an organization that holds or has applied for patents relating to the content of the manuscript?
- Do you have any other financial competing interests?
- Do you have any non-financial competing interests in relation to this paper?

If you can answer no to all of the above, write 'I declare that I have no competing interests' below. If your reply is yes to any, please give details below.

I declare that I have no competing interests

I agree to the open peer review policy of the journal. I understand that my name will be included on my report to the authors and, if the manuscript is accepted for publication, my named report including any attachments I upload will be posted on the website along with the authors' responses. I agree for my report to be made available under an Open Access Creative Commons CC-BY license (http://creativecommons.org/licenses/by/4.0/). I understand that any comments which I do not wish to be included in my named report can be included as confidential comments to the editors, which will not be published.

I agree to the open peer review policy of the journal
