# Supplementary material for: Draft genome of the Northern snakehead, Channa argus
Source: Gigascience. 2017 Mar 2;6(4):1–5. doi: 10.1093/gigascience/gix011 (PMC5530311; doi:10.1093/gigascience/gix011)
Supplement: Reviewer_2_Report_(Original_Submission).pdf [file gix011_Reviewer_2_Report_(Original_Submission).pdf]

Reviewer Report

Title: "Draft genome of the Northern snakehead, Channa argus"

Version: Original Submission **Date:** 10/13/2016

Reviewer name: Paolo Franchini

Reviewer Comments to Author:

Review for the manuscript number GIGA-D-16-00078:

In this manuscript Xu and colleagues reports the draft genome of the Northern snakehead (Channa argus), an economically important freshwater fish that is mainly distributed in Asia and Africa. However, this fish was recently released in North America where, because of its aggressive behavior, represents a dangerous invasive species. Notably, this fish has a specialized breathing organ that allows aerial breathing and thus survival for days on land. Considering its economic relevance, the particular additional breathing mode and the role in affecting ecosystem balances, the draft genome of the snakehead will be a valuable resource to facilitate future biomedical, ecological and in general research targeting phenotypic traits.

However, even though I believe that the amount of sequence data allowed to generate a robust draft genome, the authors did not analyze the genome deeply, and they did not address any specific scientific question. In the light of this main concern and of other minor points, I think that this manuscript needs a substantial revision before to be considered for publication in Gigascience, a journal that publishes robust studies relying on 'big data'.

Major comments:

Nowadays the production of million/billion sequences in not a limiting factor anymore, therefore the publication of a genome is not interesting enough without a deep data mining to provide a detailed characterization of the genome and, more importantly, to address relevant biological questions. From a physiological point of view, for example, with its bimodal breathing mode, the Northern snakehead represents a great system to investigate the gene/gens pathways related to respiratory metabolisms. Using other published genomes, with a comparative genomics approach, it would be interesting to analyze what is peculiar of this species. I believe that this type of analysis, and this is only an example, will make this study more interesting for a broader audience.

The manuscript suffers from lack of clarity. Many sentences would benefit from being re-written and in general all the manuscript should be proofread before to be resubmitted to this or to any other journal.

Minor comments:

Line 48: 86.0-Gb □ 86 Gb

Line 65: "Contrary" to what?

Lines 69-72: This sentence is poorly written, please revise it.

Line 78-90: The whole paragraph is weak as several details are missing:

- Which reagents/kit did you use for DNA extraction?
- Which Illumina kit was used to construct the genomic libraries? Did you use PCR free kit for the paired end libraries? And for the Mate pair? Did you use the Nextera kit? This information is important and the way in which the Mate pair have been processed/filtered needs to be detailed.
- I guess that the read length is 2x100 bp. Please state it.
- How many reads for paired read and how many for Mate pair reads? (translate in Gb too).
- For the further genome assembly, are short insert-reads and long-insert reads used separately in different steps? Please add more details.
- Which software and what criteria have been used to process low-quality and redundant reads?

Line 101: What are these "optimized parameters"?

Line 120: For the TE analysis, more details about other class of TEs should be provided. How many known and novel TEs were detected in the genome?

Line 112: more than 97.6% is vague. Why don't just use the number of covered CEG sequences?

Line 141-145: Why the protein sequences from Stickleback and Asian seabass, the two closest species, were not included in this analysis?

Line 163-164: The text is not consistent with the Figure 1a. In the text, the authors state that there are 963 species-specific gene families in the Northern snakehead genome, while the corresponding number, as I can see in the Venn diagram, is 24.

Level of Interest

Please indicate how interesting you found the manuscript: An article of limited interest

Quality of Written English

Please indicate the quality of language in the manuscript: Not suitable for publication unless extensively edited

Declaration of Competing Interests

Please complete a declaration of competing interests, considering the following questions:

- Have you in the past five years received reimbursements, fees, funding, or salary from an organisation that may in any way gain or lose financially from the publication of this manuscript, either now or in the future?
- Do you hold any stocks or shares in an organisation that may in any way gain or lose financially from the publication of this manuscript, either now or in the future?
- Do you hold or are you currently applying for any patents relating to the content of the manuscript?
- Have you received reimbursements, fees, funding, or salary from an organization that holds or has applied for patents relating to the content of the manuscript?
- Do you have any other financial competing interests?
- Do you have any non-financial competing interests in relation to this paper?

If you can answer no to all of the above, write 'I declare that I have no competing interests' below. If your reply is yes to any, please give details below.

I declare that I have no competing interests

I agree to the open peer review policy of the journal. I understand that my name will be included on my report to the authors and, if the manuscript is accepted for publication, my named report including any attachments I upload will be posted on the website along with the authors' responses. I agree for my report to be made available under an Open Access Creative Commons CC-BY license (http://creativecommons.org/licenses/by/4.0/). I understand that any comments which I do not wish to be included in my named report can be included as confidential comments to the editors, which will not be published.

I agree to the open peer review policy of the journal
